# AUTOMATED ASSESSMENT OF UPPER LIMB SPASTICITY IN STROKE PATIENTS WITH FUSION OF MULTICHANNEL SURFACE ELECTROMYOGRAPHY FEATURES

**DOI:** 10.2340/jrm.v57.43745

**Published:** 2025-08-28

**Authors:** Xin LI, Feiyu NONG, Xu ZHANG, Lin CHEN, Tian LI, Shengliang SHI, Yaobin LONG

**Affiliations:** 1 Department of Rehabilitation, The Second Affiliated Hospital, Guangxi Medical University, Nanning; 2 College of Mechanical Engineering, Guangxi University, Nanning; 3 Tianjin Key Laboratory of Acute Abdomen Disease-Associated Organ Injury and ITCWM Repair, Institute of Integrative Medicine of Acute Abdominal Diseases, Tianjin Nankai Hospital, Tianjin Medical University, Tianjin; 4 Department of Neurology, The Second Affiliated Hospital, Guangxi Medical University, Nanning, China

**Keywords:** stroke, spasticity, sEMG, MAS, k-NN

## Abstract

**Objective:**

The objective of this study was to investigate a more accurate and efficient technique for assessing spasticity in stroke patients via surface electromyography (sEMG).

**Methods:**

45 hemiplegic individuals were recruited and spasticity was assessed via the modified Ashworth scale (MAS). Multichannel sEMG data were collected from 3 muscles: the long head of the biceps brachii (LB), the short head of the biceps brachii (SB), and the brachioradialis (BR). Both time-domain and frequency-domain features were extracted. A K-nearest neighbour (k-NN) classifier was used to develop a new feature vector consisting of multichannel sEMG features. Finally, a model using this new feature was constructed and evaluated for classification accuracy.

**Results:**

Data from 40 patients were analysed, revealing significant correlations between MAS scores and sEMG features. Specifically, MAS exhibited strong positive correlations with 3 time-domain features: root mean square (RMS), integral sEMG (iEMG), and envelope area (EA) (*r* > 0.7). In contrast, frequency-domain features were negatively correlated with the MAS score (*r* < −0.7). A single-channel model and a single-feature model were developed as baselines. A k-NN classifier using a novel feature vector – integrating single-channel and single-feature data – enabled automatic spasticity grading, surpassing the performance of the baseline models. The proposed multichannel sEMG feature fusion model achieved an average accuracy of 78.7%, significantly outperforming both the single-channel model (LB: 66.0%, SB: 64.3%, BR: 70.4%) and the single-feature model (RMS 70.8%, iEMG 71.4%, and EA 63.4%).

**Conclusions:**

Compared with single-channel and single-feature models, the k-NN model, which uses multichannel sEMG features, has superior accuracy in spasticity assessments and is a reliable tool for objective evaluation. This approach holds promise for enhancing rehabilitation strategies by enabling precise and data-driven efficacy assessments, ultimately improving patient outcomes.

## LAY ABSTRACT

Spasticity assessment plays a critical role in the formulation of rehabilitation strategies and the evaluation of treatment outcomes in stroke patients. This study aims to identify a more accurate and efficient method for assessing spasticity in stroke patients using surface electromyography (sEMG). sEMG data were collected from 40 patients, and various spasticity quantification models were developed and compared in terms of performance. The results showed that the k-NN model based on multi-channel sEMG features achieved higher average accuracy on the test set than both the single-channel model and the single-feature classification model. In conclusion, the k-NN model provides enhanced accuracy in spasticity assessment and represents a reliable tool for objective clinical evaluation.

Stroke, comprising ischaemic stroke and haemorrhagic stroke, is a prominent global cause of mortality and enduring disability (1, 2). The incidence of stroke is expected to rise in the coming years (3, 4), mostly owing to an ageing population (5) and increasing hazardous food and lifestyle choices (6). Poststroke spasticity occurs in approximately 25% of patients within 3 days of a stroke and in 46% of patients 1 year poststroke (7, 8). Early-stage spasticity after a stroke frequently signals the start of motor function recovery. However, increased spasticity might cause side effects such as pain, stiffness, pressure sores, weakening of the muscles, joint tightness, and restricted movement. These challenges strongly affect patients’ daily tasks, impede their rehabilitation, and impose a burden on their family and society (9–12).

With the increasing prevalence of stroke, stroke spasticity therapy has attracted considerable attention. Spasticity management in stroke patients is a systematic process grounded in objective and reliable assessment outcomes, and it enables physicians to assess treatment outcomes and formulate rehabilitation plans. The Modified Ashworth Scale (MAS), Clinical Spasticity Index (CSI), rapid premature rupture of membranes (PROM), and other semiquantitative scales are used in clinical practice to assess spastic muscle tone (13–15). Among them, the MAS is the most widely used scale. Scholars have attempted to improve the reliability of MAS by standardizing the examination with regard to movement velocities and angles, and improved reliability has been demonstrated (16). Nevertheless, a lack of validity is a recognized issue for clinical evaluation because of the impact of secondary stiffness in muscle and other tissues, which inevitably contributes to resistance to passive stretching of the muscle. Therefore, a more objective and quantitative technique for evaluating spasticity is needed to guide rehabilitation and monitor the efficacy of spasticity.

Leszczyńska and Huber reported surface electromyography to be a seemingly more effective approach than clinical scale assessments for measuring spasticity and strength in people with incomplete SCI (17). Surface electromyography (sEMG) is a noninvasive diagnostic method in which electrodes are used to record the currents generated by muscle contractions on the surface of the skin. Using this technique, the dynamics of movement and the function of muscles one can be analysed (18, 19). Many researchers have investigated various assessment techniques for examining the muscular function of spastic limbs in stroke patients by using sEMG signals. According to a few studies, there is a significant difference in the sEMG parameters of spastic upper limbs between the hemiplegic and non-hemiplegic sides among stroke patients (20, 21), and the dynamic stretch reflex threshold of sEMG signals is strongly connected to the MAS score (22). In another study, a correlation between the MAS and the RMS amplitude of an sEMG signal was discovered (23). According to these studies, the degree of spasticity can be determined on the basis of the sEMG signals of spastic muscles. However, different studies have used distinct techniques to analyse sEMG signals. Because the selected sEMG eigenvalues were relatively single or studies included only those patients with MAS scores of 2 or 3, it was challenging to establish a common standard.

On this basis, this study presents a comprehensive quantitative assessment method to evaluate muscle spasticity grades in stroke patients via the fusion of multichannel sEMG features. In the proposed approach, multisource features are integrated via 3-channel sEMG signals from the long head of the biceps brachii (LB), short head of the biceps brachii (SB), and brachioradialis (BR) during passive arm flexion and extension. Specific key feature parameters, including time-domain and frequency-domain features, are obtained and analysed. These parameters are then assigned weights on the basis of the pertinent amplitudes, and the features are fused to create new vectors and realize multichannel feature fusion. The resulting fused-feature model is used to assess muscle spasticity levels in several dimensions and muscles, thereby improving the measurement accuracy and robustness of the spasticity level.

## METHODS

### Participants

This study included 45 male patients with hemiplegic stroke who were admitted to the Rehabilitation Department of the Second Affiliated Hospital of Guangxi Medical University between October 2022 and October 2023. The study was approved by the hospital’s Ethics Committee (Approval No. 2022-KY (0795)) and conducted in accordance with the Declaration of Helsinki. All patients provided informed consent before enrolment. The inclusion criteria were as follows: (*i*) aged between 18 and 80 years; (*ii*) stroke diagnosis confirmed by cranial CT or MRI, indicating cerebral haemorrhage or cerebral infarction; (*iii*) stable condition following a first-ever stroke, with onset exceeding 2 weeks; (*iv*) Mini-Mental State Examination (MMSE) score ≥ 27, ensuring the ability to follow experimental procedures; and (v) hemiplegic upper limb Brunnstrom stage ≥ II. The exclusion criteria were as follows: (*i*) unconsciousness or unstable condition; (*ii*) history of multiple strokes; (*iii*) inability to perform MAS assessment because of joint contracture, soft tissue injury, or other reasons; (*iv*) muscular dystonia induced by bilateral hemiplegia, brain trauma, multiple sclerosis, or other non-cerebrovascular accidents; and (*v*) skin damage or upper limb fractures on the affected side, preventing sEMG data collection.

### Data acquisition system

A multichannel sEMG system was used to collect data in our experiments. The default sampling frequency of the system was 500 Hz, and the maximum sampling rate was 2,000 Hz. This system was equipped with several electrode pads and a data acquisition host computer supporting simultaneous sEMG signal data acquisition from up to 6 channels. The collected sEMG data were transmitted to the computer through a signal line, and the acquired data were processed via MATLAB 2020 (MathWorks, Natick, MA, USA) and SPSS 25 (IBM Corp, Armonk, NY, USA).

### Data recording program

**Fig. 1:**
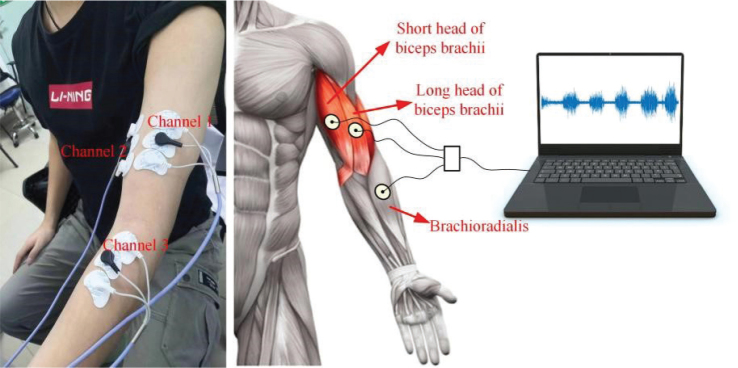
Data collection process. (A) Position of the electrode sheet and distribution of muscle positions in each channel: channel 1 corresponds to the long head of the biceps brachii (LB), channel 2 corresponds to the short head of the biceps brachii (SB), and channel 3 corresponds to the brachioradialis (BR). (B) sEMG signal data acquisition system: electrode pads, a multichannel sEMG device, and a data acquisition host computer.

The data were gathered by 2 skilled medical personnel. One person performed the MAS assessment and collected the sEMG data, while the other person supported him. Patient data were collected at the same time each day, with the patients lying down in a quiet room or relaxing in bed. Before sEMG data collection, the MAS was used to assess the patients’ degree of upper limb spasticity. The MAS rating is divided into 6 levels: 0, 1, 1+, 2, 3, and 4. The patients were then instructed to rest quietly for 30 min. Thereafter, the patients’ upper limbs were examined before the procedure to ensure that they maintained the natural position of the human body, avoiding forearm pronation or supination. The muscle belly of the patients was exposed to collect the sEMG signals, including the LB, SB, and BR. After 75% alcohol was used to disinfect the skin, electrode pads were placed on the LB, SB, and BR. Each muscle belly was covered with 3 disposable electrode pads. The sEMG signal acquisition system was configured to have a sampling frequency of 1,024 Hz. After stabilization of the baseline sEMG signal, the doctor directed passive limb motions before gathering and storing data (Fig. 1). Resting-state data were collected for 2 min to ensure baseline stability, followed by passive flexion and extension. During data collection, the clinician immobilized the patient’s elbow joint by using 1 hand, held the wrist with the other hand, and passively extended the elbow joint to elicit the stretch reflex. To prevent muscle fatigue, each flexion and extension exercise lasted approximately 10 s, and a stopwatch was used to ensure consistent speed in each test. Three consecutive sets of passive elbow flexion and extension exercises, each lasting 30 s, were performed in a cycle, and between 2 cycles, a 30-s break was provided. After the loop was completed 3 times, another 2 min was needed to collect data in the resting state. Overall, the data collection procedure lasted approximately 7 min. The patient remained in place and relaxed for 10 min after the first round of data collection. The same procedure was then followed to collect the second set of sEMG data.

### sEMG data processing

Several crucial procedures are involved in the automated quantitative assessment of spasticity grade in stroke patients. To reduce noise, the original data were first segmented and filtered to obtain clean sEMG signals. Three time-domain features, namely, the root mean square (RMS), integral sEMG (iEMG), and envelope area (EA), were subsequently retrieved from each segment of the sEMG signal data. Additionally, 2 frequency-domain properties were computed, namely, the mean power frequency (MPF) and median frequency (MF). The suggested features were normalized, and by using the physician’s MAS assessment data, a correlation analysis of the resulting feature parameters was subsequently performed.

After the aspects with poor associations were eliminated, the correlations between the suggested features and physicians’ MAS assessment results were evaluated. The sEMG feature parameters obtained from different channels were integrated, and a new feature vector was generated by allocating parameter weights on the basis of the magnitude of correlation. Ultimately, the proposed method for automated quantitative assessment of spasticity grade among stroke patients was realized by training a k-nearest neighbour (k-NN) model.

The sEMG signal data used herein were obtained from 3 channels, where channel 1 corresponded to the LB, channel 2 to the SB, and channel 3 to the BR. The sEMG signal data of each channel were divided into 3 equal segments depending on the time indices of the signals to facilitate effective time- and frequency-domain analyses of the multi-segment sEMG signal data. These segments were named Segment A, Segment B, and Segment C. Each signal segment comprised 3 passive flexion and extension sEMG data samples.

Inherent noise was introduced during sEMG signal acquisition. Although the sEMG acquisition device performs filtering via its hardware, noise such as electrocardiogram artifacts and interference from power lines might persist within the signals. To mitigate the impact of this noise on the experimental results, a 20–450 Hz bandpass filter and a 48–52 Hz notch filter were designed and used for filtering the data. A bandpass filter was used to eliminate high-frequency components in the sEMG signals, whereas a notch filter was used to eliminate low-frequency and industrial frequency components. This preprocessing step helps enhance the subsequent feature extraction and sEMG signal analysis.

### Time domain and frequency domain feature extraction

Five standard feature parameters were extracted from each segment of the sEMG signal data to elucidate the relationship between the sEMG signals and the degree of upper limb muscle spasticity in stroke patients. These features were MPF, MF, 2 frequency-domain features; and 3 time-domain features, namely, RMS, iEMG, and EA.

For a segment of the sEMG signal data *x*(*t*) of length N, the EA calculation involves several steps. Initially, all the extreme value points in the original signal were extracted. The upper and lower envelope curves (*L*(*t*) and *l*(*t*)) of the sEMG signal were subsequently obtained by fitting the extracted extreme value points to a spline curve. The areas (*EA_up_* and *EA_down_*) formed by *L*(*t*) and *l*(*t*)) with the axis were computed and summed to obtain the total envelope area *EA*.


$$EA_{up}=\left|\int_{0}^{N}L\left(t\right)\right|,EA_{down}=\left|\int_{0}^{N}l\left(t\right)\right|$$ (1)

$$EA=EA_{up}+EA_{down}$$ (2)

As the varied magnitudes had different feature parameters, those with larger values tended to dominate, potentially overshadowing the smaller contributions. The max‒min normalization method was used to align the derived feature parameters of the signals of the same order of magnitude and to guarantee comparability between them. All the feature parameters within the standardized range of (0, 1) were converted via this procedure.


$$x_{norm}=\frac{x-x_{min}}{x_{max}-x_{min}}$$ (3)

Three different channels were fused separately to find the optimal subset features. The correlation coefficients between the feature characteristics of the various channels and the MAS were used to assign weights. This helped us construct new feature vectors. If the correlation coefficients between the features (*e_1_*, *e_2_*, *e_3_*) of the LB, SB, BR, and MAS are depicted as *r_1_*, *r_2_*, and *r_3_*, after Spearman correlation analysis, the feature weight values assigned to the 3 channels are given as follows:


$$\omega_{i}=\frac{r_{i}}{r_{1}+r_{2}+r_{3}},\;(i=1,\;2,\;3)$$ (4)

This methodology helped us determine the contribution weights of the different features of each muscle channel to the MAS level. A new feature vector was subsequently generated by fusing the feature parameters obtained from the 3 channels.


$$e=\omega_{1}r_{1}+\omega_{2}r_{2}+\omega_{3}r_{3}$$ (5)

### Model construction

The feature vectors were used to build a k-NN classifier, which was used to classify the participants’ spasticity grades. The k-NN algorithm is a classic machine learning method used for classification tasks. Its core principle is based on the assumption that similar instances tend to be located close to each other in the feature space. Specifically, if a new sample is surrounded by a majority of its k nearest neighbours that belong to a particular class, it is likely to belong to that same class as well. When classifying a new, unlabelled sample, the algorithm computes the distance between this sample and all labelled samples in the dataset, identifies the k closest neighbours, and then performs a majority voting mechanism on the basis of their class labels. The class with the highest number of votes among these k neighbours is assigned as the predicted class for the new sample (24). A training set and a test set were created from the 200 sample datasets at a ratio of 9:1. Within the training set, 36 sample datasets were selected for each spasticity grade, and the remaining 4 datasets were used as test sets. The classification accuracy of the proposed method was assessed by comparing the classification results obtained on the test set with the actual MAS results determined by a physician. The model was deemed able to categorize the subjects’ spasticity grades accurately if the classification results obtained via the test set agreed with the actual MAS results determined by the physician. Otherwise, the classification was deemed incorrect.

### Model hyperparameter selection

K value selection strongly affects k-NN model outcomes. In this study, 10-fold cross-validation was used to optimize this parameter. The 200 sample datasets were split into 10 equal subsets. In each iteration, 1 subset was designated as the test set, and the other 9 subsets were included in the training set. The method was iterated 10 times, and various K values were used to statistically examine the categorization accuracy rates. In step 2, the K value increased progressively from 1 to 13. Interestingly, the average accuracy tended to increase first and then decrease, peaking at 79% for K = 5. Consequently, K = 5 was deemed the best K value, meaning that the 5 points closest to a forecast point determine the category to which the point belongs.

## RESULTS

### General patient information and initial outcomes

A cohort of 45 male patients who experienced spasticity within the elbow flexor muscles of the hemiplegic upper limb because of stroke were recruited from the inpatient and outpatient departments. In the preliminary analysis, the data of 40 patients were included after 5 invalid data points were eliminated. The final sample consisted of 40 patients aged 53.6 ± 15.1 years, representing a range of MAS grades (0, 1, 1+, 2, and 3), which are summarized in Table I. Each patient underwent 2 trials, resulting in 80 data collection trials. The data from each trial were divided into 3 equal segments, resulting in 240 sets of sample data. To obtain a balanced representation covering all spasticity grades, 40 sample datasets were selected for each of the 5 spasticity grades that were collected.

**Table I:** Demographics of the patients who participated in this study

MAS	Patients, *n*	Age (years)	Course of disease (days)	Side of hemiparesis (left/right)	Type of stroke (ischaemic/haemorrhagic)
MAS 0	9	64.7 ± 8.8	55.8 ± 35.2	4/5	6/3
MAS 1	8	54.6 ± 12.6	61.1 ± 34.6	4/4	3/5
MAS 1+	8	51.1 ± 10.3	48.4 ± 32.5	5/3	2/6
MAS 2	8	50.1 ± 16.3	47.6 ± 29.7	5/3	3/5
MAS 3	7	44.9 ± 21.3	46.9 ± 20.2	2/5	2/5

### Correlation and influence weights of the MAS and sEMG features

The results indicated that MAS and 3 time-domain features, namely, RMS, iEMG, and EA, were positively correlated. In contrast, MAS and 2 frequency-domain features, namely, MF and MPF, were negatively correlated. The correlation coefficients of the time-domain features (*r* > 0.7) were greater than those of the frequency-domain features (*r* < –0.7). Consequently, the 2 frequency-domain features, MPF and MF, were excluded from the original feature set. In this manner, by retaining some of the time-domain features that exhibited strong associations, the dataset was simplified, and algorithmic efficiency was increased (Table II).

**Table II:** Spearman’s correlation coefficients of the sEMG features and weights of influence on the MAS

Item	Time-domain features’ correlations & weights			Frequency-domain features’ correlations	
	RMS	iEMG	EA	MPF	MF
LB	0.80 (0.33)	0.71 (0.32)	0.77 (0.32)	–0.41	–0.31
SB	0.83 (0.34)	0.79 (0.36)	0.80 (0.37)	–0.65	–0.54
BR	0.78 (0.32)	0.73 (0.33)	0.81 (0.34)	–0.49	–0.55

### Classification results

The training results were obtained after 10-fold cross-validation, and a confusion matrix was used to assess the relationship between the predicted and true values. The correctly predicted cases were represented by the values on the diagonal line that ran from the upper left corner to the lower right corner of the matrix. Conversely, the values at other positions corresponded to incorrectly predicted instances. The classification effect of the classifier was enhanced using a more significant number and a darker colour for this diagonal line. An analysis of the confusion matrix revealed that the majority of the predicted samples exhibited a prediction deviation of 1 or less, with the exception of a small number of samples in the MAS 1 and MAS 1+ levels, which exhibited a prediction deviation of 2 in incorrect classification cases.

The test data prediction outcomes for each MAS level were tabulated individually (Table III). The following metrics were computed for a specific spasticity level: true positive (TP), which denotes the number of samples predicted correctly at a given level. A false negative (FN) represents the number of samples at a given level that are misclassified to a different level. A false positive (FP) indicates the number of samples that are predicted at a given level but actually belong to a different level.

**Table III:** Classification performance and classification results of the multichannel sEMG feature fusion model

Item	MAS 0	MAS 1	MAS 1+	MAS 2	MAS 3
TP	37	33	30	25	33
FN	3	7	10	15	7
FP	0	6	22	11	3
Precision	1.00	0.84	0.57	0.69	0.91
Recall	0.92	0.82	0.75	0.63	0.83
F1-Score	0.96	0.83	0.65	0.65	0.86

**Fig. 2:**
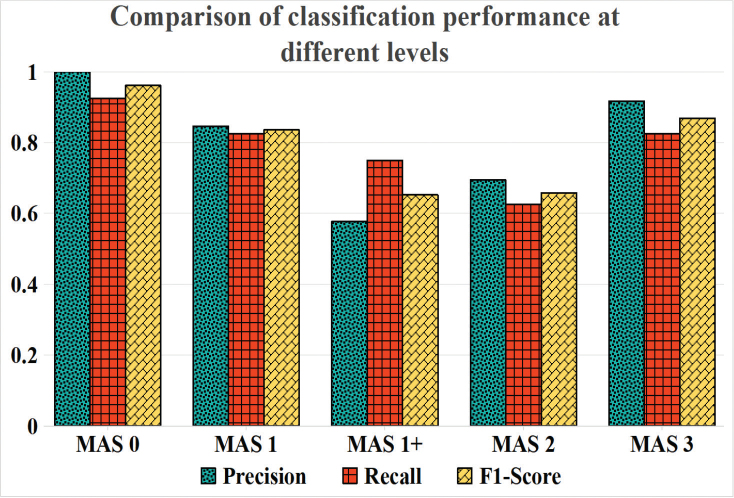
Comparison of classification performance at different MAS levels. The X-axis represents the MAS level, and the Y-axis represents the precision, recall, and F1 score metrics.

The classification performance of the proposed classifier for each MAS level was evaluated via the metrics precision, recall, and F1 score (Fig. 2). As indicated in Table IV, the best classification metrics were obtained for MAS 0 (F1 score = 0.96) and MAS 3 (F1 score = 0.86), with precision and recall rates of 100% and 92% for MAS 0, and 91% and 83% for MAS 3. Acceptable performance was obtained for MAS 1 (F1 score = 0.83), with recall and precision rates of 82% and 84%, respectively. However, in categorizing MAS 1+ and MAS 2, the classifier performed suboptimally (F1 score < 0.7).

**Table IV:** Comparison of different categorization models’ classification results

Models	Single channel model			Single feature model			Multi feature model
Channel and feature	LB	SB	BR	RMS	iEMG	EA	Multichannel feature fusion
Accuracy	66.0%	64.3%	70.4%	70.8%	71.4%	63.4%	78.7%

To evaluate the superiority of the multichannel sEMG feature classification model, we developed a single-channel model and a single-feature model. Thereafter, we developed a multi-feature fusion model based on the 2 aforementioned models and compared the 3 models. The 3 time-domain features (RMS, iEMG, and EA) of the unfused sEMG signals from each channel were used in the single-channel classification model. In contrast, the 3 time-domain features of the unfused sEMG signals from each channel were used in the single-feature classification model. Each model underwent 10-fold cross-validation to secure the classification results. The average accuracy metric over 10 tests was used to evaluate the prediction effectiveness of each model. In each test, different models were applied to the same test dataset, and accuracy was defined as the ratio of correctly classified samples to the total number of samples. As seen in Table IV, the average accuracy of the multi-feature fusion classification model is 78.7%, which represents a substantial improvement over the accuracy of the single-channel model (LB 66.0%, SB 64.3%, and BR 70.4%) and single-feature classification model (RMS 70.8%, iEMG 71.4%, and EA 63.4%).

## DISCUSSION

sEMG is a prevalent diagnostic and treatment tool in clinical research (25), and the features of sEMG signals can effectively reflect changes in muscle strength (26) and decode categories and goals of movement (27–29). In our previous research, we used a single-channel sEMG device to quantitatively evaluate the relationship between the gastrocnemius and MAS in stroke hemiplegia patients. The results indicated that sample entropy and MAS were correlated (*r* = 0.745) and that there was a linear correlation between the sample entropy of sEMG and the MAS grade (30). However, accurately evaluating muscle tone is inherently difficult because patients’ entropies differ greatly. On the basis of these findings, we propose a technique to automatically and quantitatively assess the level of upper limb muscle spasticity in stroke patients by using multichannel sEMG data in this study.

Our findings validated that sEMG can effectively reflect spasticity levels in stroke patients, which was consistent with the results of other studies (23, 31). Yu et al. obtained sEMG signals from the upper limbs of 15 stroke patients who experienced elbow flexor spasticity. Their results revealed a significant difference between the spastic upper limb RMSs of sEMG between the hemiplegic and non-hemiplegic sides of stroke patients (23). In addition, our results revealed positive correlations between the MAS score and 3 time-domain features (RMS, iEMG, and EA). In contrast, the MAS and the 2 frequency-domain features (MF and MPF) were negatively correlated. The correlation coefficients of the time-domain features (*r* > 0.7) were greater than those of the frequency-domain features (*r* < –0.7). This finding was consistent with the findings of Yu et al. (23), who reported that the MAS score was positively correlated with the peak RMS of the paretic BR (*r* = 0.59) and with the mean RMS of the paretic biceps and BR (*r* = 0.62, *r* = 0.74), but it was not significantly correlated with the peak RMS of the paretic biceps (*r* = 0.49). Although we found a stronger correlation between the MAS score and RMS (*r* ≥ 0.78) than that reported by Yu et al., we were unable to rule out the influence of variables such as different collection methods, equipment, and sample sizes.

A few unique findings of this study are as follows. An analysis of the confusion matrix revealed that the majority of the predicted samples exhibited a prediction deviation of 1 or less, with the exception of a small number of samples in the MAS 1 and MAS 1+ levels, which exhibited a prediction deviation of 2 in cases of incorrect prediction. Moreover, the classifier performed suboptimally for the MAS 1+ and MAS 2 levels in terms of differentiating between these levels. These results were similar to a few of the results obtained in our previous study, in which we discovered that sample entropy varied little between MAS 1 and MAS 1+ or between MAS 1+ and MAS 2. We consider MAS 1+ to be the central level between levels 1 and 2, and there are no clear boundaries between levels 1 and 1+ or between 1+ and 2. This may also be because the sample size was small, resulting in errors in the doctor’s subjective judgement and patient emotional stress. Moreover, we considered the correlations among patient age, sebum thickness, and other factors, such as resistance and noise, during the process (32). In future studies, we will increase the sample size and improve the experimental operating procedures. Furthermore, our research indicated that the average accuracy of the multichannel fusion model on the test set reached 78.7%. Compared with single-channel and single-feature models, the k-NN model using multichannel sEMG features demonstrates superior accuracy in spasticity assessments. This approach holds promise for enhancing rehabilitation strategies by enabling precise and data-driven efficacy assessments.

Spasticity not only affects patients’ functional recovery but also significantly impairs their ability to perform daily living activities. Clinical rehabilitation assessments often integrate multiple standardized scales for comprehensive evaluation. sEMG offers several advantages, including strong repeatability, broad detection capabilities, and the ability to monitor muscle activity during exercise, making it a promising tool for the clinical evaluation and diagnosis of neuromuscular diseases (33, 34). This evaluation method is also expected to become a method for evaluating other functions, combined with several commonly used and reliable assessment scales, such as the Fugl-Meyer assessment (FMA), functional independence measure (FIM), Brunnstrom stage, and activities of daily living (ADL). Researchers have identified representative sEMG monitoring sites that reflect forearm muscle activity during simulated ADL tasks (35). Additionally, studies have collected sEMG signals from lower limb muscles under different daily activity conditions and demonstrated that sEMG features can effectively distinguish and classify lower limb movement patterns (36). A significant correlation has also been observed between sEMG signal characteristics and the Brunnstrom recovery stages in stroke patients (37). The integration of sEMG with rehabilitative medicine and mechanical engineering represents a promising direction for future research (38–40). In our subsequent research, we plan to conduct multiregional, multicentre collaborative studies, expand the sample size, and incorporate diverse assessment techniques to enable a more comprehensive evaluation of spasticity from multiple perspectives, thereby establishing a solid theoretical foundation for more accurate rehabilitation strategies. In the future, we intend to combine multiple functional assessment modalities and apply various sEMG analysis methods to holistically evaluate patient status, aiming to develop a more comprehensive and convenient objective assessment approach.

### Limitations

There are several limitations in this study. It gathered sEMG data only from male patients and has a small sample size. Second, this study primarily collected electromyographic data from hemiplegic upper limbs and did not compare or assess data from hemiplegic lower limbs or healthy limbs. Furthermore, the study included only spastic muscles from stroke patients; therefore, the sample sources were not broad, even though spasticity can be an indication of many different diseases. The equipment used for collection is relatively simple. To provide new ideas for accurate rehabilitation, spastic muscles in different limbs can be thoroughly evaluated via a range of evaluation tools, and the sample size and patient range can be increased in the future.

### Conclusions

Compared with single-channel and single-feature models, the k-NN-based multichannel sEMG feature fusion model provides more valuable insights into the development of logical rehabilitation programs with accurate efficacy assessments. In the future, researchers need to conduct multiregional, multicentre collaborative research and increase the sample size to evaluate limb spasticity more accurately.

## Data Availability

The dataset may be found at: https://www.jianguoyun.com/p/DQQZNuEQuaiFChj5iO4FIAA
